# Discussing some basic critique on Journal Impact Factors: revision of earlier comments

**DOI:** 10.1007/s11192-012-0677-x

**Published:** 2012-02-29

**Authors:** Thed van Leeuwen

**Affiliations:** CWTS, Leiden University, Willem Einthoven Gebouw, Wassenaarseweg 62a, PO Box 905, 2300 AX Leiden, The Netherlands

**Keywords:** Journal Impact Factor, Length of citation windows, Document types, Journal Subject Categories

## Abstract

In this study the issue of the validity of the argument against the applied length of citation windows in Journal Impact Factors calculations is critically re-analyzed. While previous studies argued against the relatively short citation window of 1–2 years, this study shows that the relative short term citation impact measured in the window underlying the Journal Impact Factor is a good predictor of the citation impact of the journals in the next years to come. Possible exceptions to this observation relate to journals with relatively low numbers of publications, and the citation impact related to publications in the year of publication. The study focuses on five Journal Subject Categories from the science and social sciences, on normal articles published in these journals, in the 2 years 2000 and 2004.

## Introduction

In earlier studies, criticism on Journal Impact Factors was centered around a number of key problems when it comes to the famous Journal Impact Factors, produced annually by Thomson Reuters in their Journal Citation Reports. Criticism was of a various nature, on the one hand focused on the mathematical issues related to the calculation of Journal Impact Factors, and the somewhat unclear issue of the concept ‘citeable item’ (see Moed and van Leeuwen 1995; Moed and van Leeuwen^ 1996^), while criticism of a more methodological nature centered around three different topics. The first related to the issue of the lack of proper field normalization when it comes to Journal Impact Factors, which makes it difficult if not impossible to make direct comparisons between Journal Impact Factors values between two or more so called Journal Subject Categories. The second methodological critique on Journal Impact Factors was somewhat related to the topic of citeable items, as the Journal Impact Factors do not take into consideration the composition of a journal in terms of its documents, resulting in journal listings in the Journal Citation Reports in which journals that contain only or many reviews dominate the rankings in the respective Journal Subject Categories. A final methodological critique on the Journal Impact Factors evolved around the issue of the length of the applied citation window. Main issue here was the too short period of citation impact measurement of 1–2 years, which was considered as too short (van Leeuwen et al. 1999; Vanclay 2009, 2012). Although Thomson Reuters nowadays works with Journal Impact Factors with longer windows, the most often used one is the Journal Impact Factor with the short windows.

This paper will deal with this latter issue mainly, trying to review the earlier position taken by the author in various publications. In earlier publications the main focus was on the issue of the citation history reaching a citation peak moment. This approach was based upon the separate years within citation impact measurement rather than on a cumulative approach. The analysis showed that in almost all fields we analyzed, the peak moment of citation impact measurement was observed well beyond the period of 2 years, with only Biochemistry and molecular biology as a field in which the peak moment of citation impact was on average close to this 2 year length (as applied in the calculation of Journal Impact Factors, see van Leeuwen et al. 1999). So the question that was raised in the previous studies on the length of the citation windows when it comes to citation impact measurement underlying the calculation of Journal Impact Factors was: within this short time frame of 1–2 years after publication, what part of citation impact do you measure, and is the applied length of the citation window long enough? As stated above, Thomson Reuters started supplying Journal Impact Factors based on longer citation windows, so this criticism was taken up seriously. In this paper we will review the previously taken critical position, in particular the conceptual approach of the criticism on the validity of the applied length of citation windows (van Leeuwen et al. 1999).

In this paper we will apply an analysis on the citations related to a cumulative-based impact measurement of journals in five Journal Subject Categories, namely Biochemistry and molecular biology, Mathematics, and Pathology in the sciences, and Economics and Information and library sciences in the social sciences domain. Furthermore, our analysis will only use normal articles published in these journals.

## Research background

As stated above, criticism on Journal Impact Factors focused on a number of problems, one of it of a more mathematical/numerical nature, and three of a more methodological/conceptual nature. Here these are summarized:

Mathematical:

- The problem of the unequal contents of the nominator and the denominator, thereby creating the problem of ‘citations for free’, by inclusion in the calculation of citations towards document types that are not part of the calculation (e.g., the inclusion of references towards letters, meeting abstracts, editorials, while these documents are not included in the formula of Journal Impact Factors, Moed and van Leeuwen 1995; Moed and van Leeuwen 1996).

Methodological/conceptual:

- Journal Impact Factors are not normalized towards the field they are attributed to, which causes the absolute values of Journal Impact Factors to become actually incomparable (e.g., the Journal Impact Factors ranking on top in Journal Subject Categories in biomedicine tend to outscore Journal Impact Factors in the natural sciences, while these journals outscore the journals in the social sciences. This phenomenon is a mere representation of the citation cultures in these various domains (Vinkler 1991; Ugolini et al. 1997a; Ugolini et al. 1997b; van Leeuwen and Moed 2001). As such, Journal Impact Factors are highly problematic when direct comparison across fields is applied, particularly in an evaluative context (van Leeuwen and Moed 2002)).
- Journal Impact Factors are not normalized when it comes to the composition of a journal in terms of the document types published in the journal. This causes the journals that contain many review papers to outscore journals that contain a variety of document types. This is again a reflection of the citation culture that relates to citing reviews (van Leeuwen and Moed 2004). Yet another problem related to reviews in the Web of Science is the classification of these documents itself, as it seems that this is not done in a consistent and valid way, thus creating a rather heterogeneous class of documents (e.g., publications that contains certain words, such as review in either title or abstract are classified as review, while also the length of the reference list is a determining factor in the classification of documents as reviews by Thomson Reuters (Harzing 2010).
- Finally, the problem of the length of the applied citation window. As the formula of the Journal Impact Factor, at least the classical version, dictates a citation window of 1–2 years, that is, the years t-1 and t-2 (Garfield 1976). This short window of counting citation impact was considered to be disadvantageous for these fields in which citation impact starts to increase after a somewhat longer period, due to the nature of the research conducted in these fields, e.g., the laboratory-based research in biomedicine and the natural sciences, contrary to more clinical-practice or application oriented technical research as well as the social sciences (Moed et al. 1998; van Leeuwen et al. 1999; Vanclay 2009).

## Objective and research question

This paper will deal with this latter issue mainly, trying to review the earlier position taken by the author in various publications. In earlier publications the main focus was on the issue of the citation history reaching a citation peak moment. This approach was based upon the separate years within citation impact measurement rather than on a cumulative approach. The analysis showed that in almost all fields we analyzed, the peak moment of citation impact measurement was observed well beyond the period of 1–2 years, with only Biochemistry and molecular biology as a field in which the peak moment of citation impact was on average close to this 2 year length (as applied in the calculation of Journal Impact Factors). So the question that was raised in the previous studies on the length of the citation windows when it comes to citation impact measurement underlying the calculation of Journal Impact Factors was: within this short time frame of 1–2 years after publication, what part of citation impact do you measure, and is the applied length of the citation window long enough ? As stated above, Thomson Reuters started supplying Journal Impact Factors based on longer citation windows, so this criticism was taken up seriously. In this paper we will review the previously taken critical position, in particular the conceptual approach of the criticism on the validity of the applied length of citation windows.

## Data and methodology

Data used for the analysis are retrieved from the in-house version of the Web of Science at CWTS. The publications used are aggregated to the level of journals and Journal Subject Categories. Citation data in this study are based on citation linking algorithms applied in the in-house version of the Web of Science at CWTS. The selected Journal Subject Categories are Biochemistry and molecular biology, Economics, Information and library sciences, Mathematics, and Pathology. The data in this study involve two publication years, 2000 and 2004. For reasons of clarity, we only used normal articles in the analysis, thereby excluding any negative distorting effects of letters and reviews as document types.

The analysis is based on database years, both for the publications as well as the citations linked to publications. So when talking about impact in year 1, we indicate the citation impact in the year of publication, in the case of the first year analyzed in this study, database year 2000, while the impact in year 2, we indicate the citation impact in database year 2001.

For every journal in the Journal Subject category we calculated for the years 2000 and 2004 the cumulative citation impact of the normal articles. So for the year 2000 we had citation impact measured for eleven years, and seven years for 2004 (in both cases up until 2010, due to the range of the database at the moment of analysis, covering the period 1981-2010). For a proper comparison, the analysis focused on the first seven years after publication, as this period is available for both publication years. This means for the publications of 2000, we measured citation impact from 2000 up until 2006, while for the 2004 publications we used the citations up until 2010.

Next, as the journals do not all contain equally many publications on an annual basis, we grouped the journals per Journal Subject Category in a number of classes of “publications per year”, actually journal volume classes. Main principle was the construction of more or less equally large classes, preferably five, but four or six is also allowed. This is constructed similarly for both years 2000 and 2004, however, for reasons of comparability we decided to apply the same distribution on both years, with 2000 as the base year. As a side effect, we created some insight in the changes in time of the volume of the Journal Subject Categories and the classes distinguished in these classes.

The analysis conducted to answer the research question is mainly based on a comparison per class, of the positions based on citation impact of the journals involved. Per journal class based on volume of publications, Pearson correlations are calculated for the comparison of the impact in year-1 (year of publication) with year-2 (year of publication + 1), next the comparison of the impact in year-2 (year of publication + 1) with year-3 (year-2 + 1), etc. The correlations per class based on cumulative citation impact form the core of the data resulting from the analysis.

## Results

In this section the main findings of the study are presented on the level of the five Journal Subject Categories analyzed. Before getting into the details on the level of journals classes in these Journal Subject Categories, some basic data on the level of the categories are presented first. Table 1 contains an overview of the total number of journals covered in the five selected categories, the total number of publications involved, and the average number of normal articles per journals, for both 2000 and 2004.

**Table 1:** Overall contents of the five selected Journal Subject Categories, 2000 and 2004

	2000			2004		
	Nr Jnls	Nr Pubs	Average Nr Pubs	Nr Jnls	Nr Pubs	Average Nr Pubs
Biochemistry and molecular biology	238	47,346	198.9	235	43,574	185.4
Economics	184	7,698	41.8	187	7,973	42.6
Information and library science	58	1,722	29.7	57	1,867	32.8
Mathematics	170	13,304	78.3	191	14,082	73.7
Pathology	66	6,237	94.5	65	5,501	84.6

Table 1 clearly shows the differences between the five categories selected for the study, with Information and library science and Pathology as the somewhat smaller categories. Biochemistry and molecular biology and Economics are two larger categories, both composed rather heterogeneously (for the field of economics, see van Leeuwen and Calero Medina 2012). Yet another important distinguishing characteristic in the set of selected Journal Subject Categories is the large quantity of publications in Biochemistry and molecular biology (with 47.346 normal articles in 2000, and 43.574 normal articles in 2004). The high average number of publications per journal is thus to be expected, although the field contains in 2000 four journals together producing over 10.000 normal articles, and one (Journal of Biological Chemistry) with 5.486 normal articles, while in 2004 the field contains five journals with over 1,000 normal articles each, together containing 12.186 normal articles, and one journal (Journal of Biological Chemistry) with 6.156 normal articles in 2004. A final remark relates to the increase of the number of journals processed for the Journal Subject category of Mathematics (increasing form 170 to 191 journals, an increase of 12%).

In Table 2, we present the composition of the five selected Journal Subject Categories through the composed journal volume classes. For each Journal Subject Category, we created a distribution of the total number of publications of a journal in roughly five classes. In practice, this resulted in either four classes (Pathology), five classes (Information and library science), or six classes (Biochemistry and molecular biology, Economics, and Pathology). In general, the first journal volume class, which starts with journals that contain only 1 normal article, up to a value that limits the first class, is less robust. These low numbers of normal articles can be explained by either the choice for the selection of only normal articles (which excludes the reviews in review journals, thus producing journals with low numbers of normal articles), or by the fact that the Web of Science nowadays contains more journals which are indexed on a topic basis rather than a cover-to-cover basis.

**Table 2:** Contents of the five selected Journal Subject Categories, 2000 and 2004

	2000				2004			
	Nr Jnls	Nr Pubs	Average # publs.	Range of # publs.	Nr Jnls	Nr Pubs	Average # publs.	Range of # publs.
Biochemistry and molecular biology								
1–50	77	2,153	28.0	1–50	68	2,039	30.0	1–48
51–100	56	4,125	73.7	52–99	55	4,018	73.1	52–97
101–150	38	4,550	119.7	101–149	37	4,608	124.5	101–144
151–200	23	4,550	197.8	153–196	24	4,112	171.3	151–194
201–250	14	3,216	229.7	204–250	14	3,194	228.1	203–248
251–	30	22,957	765.2	255–5,486	37	25,603	692.0	255–6,156
Economics								
1–20	36	485	13.5	5–20	35	471	13.5	5–20
21–30	55	1,372	24.9	21–30	44	1,153	26.2	21–30
31–40	30	1,046	34.9	31–40	38	1,348	35.5	31–40
41–50	19	868	45.7	41–50	22	1,002	45.5	41–50
51–75	24	1,494	62.3	52–75	31	1,893	61.1	51–74
76–	20	2,433	121.7	76–236	17	2,106	123.9	76–263
Information and library science								
1–15	15	145	9.7	4–14	10	107	10.7	4–14
16–25	12	233	19.4	16–25	19	392	20.6	16–25
26–35	16	478	29.9	26–34	8	248	31.0	26–34
36–50	9	369	41.0	36–46	13	546	42.0	36–46
51–	6	497	82.8	51–108	7	574	82.0	51–143
Mathematics								
1–30	30	605	20.2	6–30	41	792	19.3	7–28
31–45	37	1,371	37.1	31–45	46	1,766	38.4	31–45
46–60	36	1,928	53.6	46–60	28	1,477	52.8	46–60
61–100	30	2,405	80.2	61–98	44	3,370	76.6	61–100
101–150	22	2,572	116.9	104–150	15	1,799	119.9	103–148
151–	15	4,423	294.9	159–479	17	4,878	286.9	160–590
Pathology								
1–35	13	340	26.2	8–33	13	285	21.9	1–33
36–70	20	910	45.5	37–62	24	1,162	48.4	36–69
71–150	17	1,675	98.5	72–146	18	1,912	106.2	71–147
151–	16	3,312	207.0	154–427	10	2,142	214.2	164–379

In Tables 3 and 4 we present the actual correlations of the comparison of the year to year impact scores per journal class. Table 3 contains the results for the publication year 2000, while Table 4 contains similar results for 2004.

**Table 3:** Year to year correlations for impact scores per journal class in five Journal Subject Categories, 2000

Journal class	y1–y2	y2–y3	y3–y4	y4–y5	y5–y6	y6–y7
Biochemistry and molecular biology						
1–50	0.85	0.93	0.99	0.99	1.00	1.00
51–100	0.91	0.99	1.00	1.00	1.00	1.00
101–150	0.96	0.99	1.00	1.00	1.00	1.00
151–200	0.99	1.00	1.00	1.00	1.00	1.00
201–250	1.00	1.00	1.00	1.00	1.00	1.00
251–	0.97	1.00	1.00	1.00	1.00	1.00
Economics						
1–20	0.62	0.97	0.98	0.99	1.00	1.00
21–30	0.74	0.94	0.98	0.99	0.99	1.00
31–40	0.28	0.94	0.98	0.99	0.99	1.00
41–50	0.85	0.99	1.00	1.00	1.00	1.00
51–75	0.86	0.97	1.00	1.00	1.00	1.00
76–	0.84	0.99	1.00	1.00	1.00	1.00
Information and library science						
1–15	0.46	0.74	0.95	0.99	0.97	0.99
16–25	0.85	0.84	0.96	0.99	1.00	0.99
26–35	0.01	0.87	0.95	0.98	0.99	0.99
36–50	0.86	0.91	0.98	0.99	0.99	1.00
51–	0.94	0.90	0.98	0.99	0.99	1.00
Mathematics						
1–30	0.83	0.95	0.96	0.99	0.99	1.00
31–45	0.72	0.95	0.99	0.99	1.00	1.00
46–60	0.79	0.97	0.99	1.00	1.00	1.00
61–100	0.79	0.96	0.99	0.99	1.00	1.00
101–150	0.87	0.99	0.99	1.00	1.00	1.00
151–	0.85	0.99	0.99	1.00	1.00	1.00
Pathology						
1–35	0.90	0.88	0.97	0.99	1.00	1.00
36–70	0.89	0.99	1.00	1.00	1.00	1.00
71–150	0.70	0.99	1.00	1.00	1.00	1.00
151–	0.98	1.00	1.00	1.00	1.00	1.00

**Table 4:** Year to year correlations for impact scores per journal class in five Journal Subject Categories, 2004

Journal class	y1–y2	y2–y3	y3–y4	y4–y5	y5–y6	y6–y7
Biochemistry and molecular biology						
1–50	0.81	0.99	0.99	1.00	1.00	1.00
51–100	0.89	0.99	1.00	1.00	1.00	1.00
101–150	0.99	1.00	1.00	1.00	1.00	1.00
151–200	0.96	0.99	1.00	1.00	1.00	1.00
201–250	0.99	1.00	1.00	1.00	1.00	1.00
251–	0.98	1.00	1.00	1.00	1.00	1.00
Economics						
1–20	0.54	0.90	0.97	0.99	1.00	1.00
21–30	0.89	0.96	0.99	0.99	1.00	1.00
31–40	0.76	0.97	0.99	0.99	1.00	1.00
41–50	0.62	0.97	0.99	1.00	1.00	1.00
51–75	0.89	0.98	0.99	1.00	1.00	1.00
76–	0.65	0.94	0.99	0.99	1.00	1.00
Information and library science						
1–15	0.72	0.86	0.97	0.99	1.00	1.00
16–25	0.20	0.88	0.97	0.99	1.00	1.00
26–35	0.98	0.83	0.95	1.00	1.00	1.00
36–50	0.69	0.88	0.98	0.99	0.99	1.00
51–	0.92	0.98	0.99	0.99	1.00	1.00
Mathematics						
1–30	0.84	0.97	0.98	0.98	1.00	1.00
31–45	0.78	0.96	0.98	0.99	0.99	1.00
46–60	0.69	0.96	0.99	1.00	1.00	1.00
61–100	0.86	0.98	0.99	0.99	1.00	1.00
101–150	0.92	0.97	1.00	1.00	1.00	1.00
151–	0.83	0.95	0.98	1.00	1.00	1.00
Pathology						
1–35	0.76	0.97	0.99	1.00	0.99	1.00
36–70	0.79	0.97	0.99	1.00	1.00	1.00
71–150	0.83	0.99	1.00	1.00	1.00	1.00
151–	0.97	1.00	1.00	1.00	1.00	1.00

**Fig. 1:**
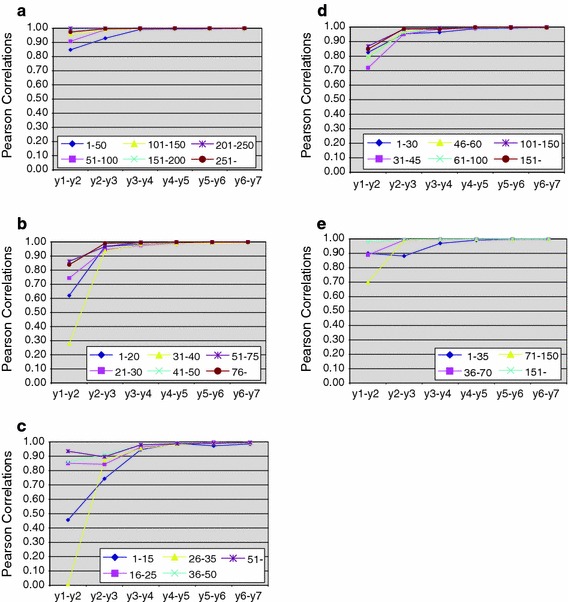
**a** Year to year correlations for impact scores per journal class in Biochemistry and molecular biology, 2000. **b** Year to year correlations for impact scores per journal class in Economics, 2000. **c** Year to year correlations for impact scores per journal class in Information and library science, 2000. **d** Year to year correlations for impact scores per journal class in Mathematics, 2000. **e** Year to year correlations for impact scores per journal class in Pathology, 2000

In Table 3 it becomes immediately clear that tow different elements are of importance in this analysis. This is clearly illustrated in the Fig. 1a–e, which are the graphical representations of the data in Table 3. A first observation is related to the relatively low values of the Pearson correlations measured form year-1 to year-2. This suggests that citation impact measurement in the first year of existence of scientific literature is very tricky and may easily lead to distortions in outcomes of citation impact measurements (which is actually the main reason for exclusion of the most recent publications in the recently launched indicator MNCS, Waltman et al. 2011), and becomes meaningful in the year after publication, as can be concluded from the strong increase in Pearson correlations in the comparison of year-2/year-3 with year-1/year-2.

Yet another important observation from the data shown in Table 3 relates to the journal class which contains the lowest number of publications annually. Although the Pearson correlation still follow an increasing pattern, the values of the correlations remain relatively lower compared to the other journal classes. In general we observe the classes with the journals with a larger quantity of publications annually to show stable patterns, of increasing similarity between the years compared.

Overall we can conclude that, except for the year-1/year-2 comparison and the journal class with the journals containing the lowest quantity of publications per year, impact increases constantly. In Biochemistry and molecular biology, the class with the lowest number of publications is somewhat deviant from the general pattern observed among the other classes, while the two journal classes with the lowest number of publications per year display the largest difference in the comparison of year-1/year-2 and year-2/year-3 (see Fig. 1a). In Economics (Fig. 1b), Information and library science (Fig. 1c), and Pathology (Fig. 1e), the main focus is on the difference between year-1/year-2 comparison with year-2/year-3, in which we find strong increases, while the correlations in year-1/year-2 are rather variable, and show strong fluctuations between journal volume classes. For Mathematics (Fig. 1d), we observe a pattern somewhat in between Biochemistry and molecular biology on the one hand, and the other three fields on the other hand. Overall we can conclude that for the publication year 2000, the correlations calculated for journal rankings within their class shows an increase with the lengthening of the citation measurement period.

In Table 4, the scores for the five Journal Subject Categories are displayed, similarly like the data in Table 3. Please note that the journal volume classes are defined similarly to that in Table 3.

Table 4 clearly shows a repetition of the observations we made for the data presented in table 3. In general, the comparison of positions for journals per journal volume class between year-1 to year-2 shows relatively low and fluctuating correlations. Comparing this first block of correlations (year-1/year-2) with the next block (year-2/year-3) clearly shows for all five Journal Subject Categories and the journal volume classes therein, increases in observed Pearson correlation scores. Next, we also notice that the journal volume class which contains journals with the lowest number of publications annually, displays the lower correlations scores, while the other classes, containing journals with more publications per year display earlier (that is, shorter after the moment of publishing) higher correlation scores.

**Fig. 2:**
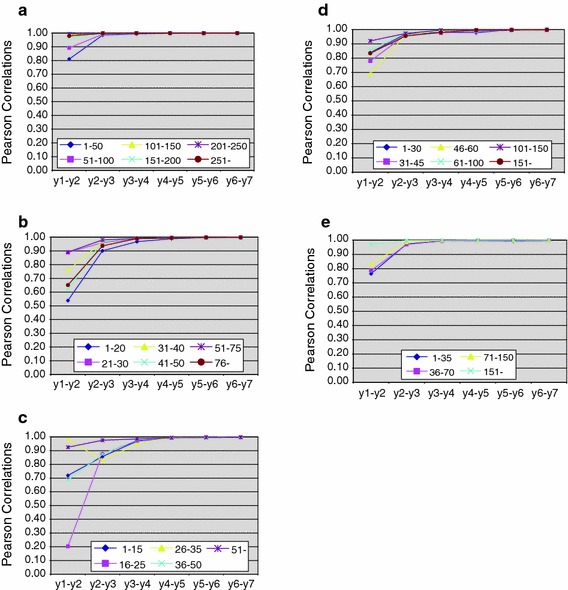
**a** Year to year correlations for impact scores per journal class in Biochemistry and molecular biology, 2004. **b** Year to year correlations for impact scores per journal class in Economics, 2004. **c** Year to year correlations for impact scores per journal class in Information and library science, 2004. **d** Year to year correlations for impact scores per journal class in Mathematics, 2004. **e** Year to year correlations for impact scores per journal class in Pathology, 2004

For Biochemistry and molecular biology (Fig. 2a) we observe that only the rank correlations in the comparison of the journal impact levels between year-1 and year-2 are relatively low (even somewhat lower as compared to the publications from 2000), but the Pearson correlations for the year-2/year-3 comparison are higher for 2004 as compared to 2000, and keep increasing whenever the citation measurement becomes longer. For Economics (Fig. 2b), we observe the Pearson correlations to be more closes as compared to the publications from 2000. The year-1/year-2 comparison fluctuates between 0.54 and 0.89. However, the comparison of the year-2/year-3 correlations displays a more close range of scores (0.90 to 0.98). In the next comparisons, the range of Pearson correlations becomes even more close. In Fig. 2c, displaying the scores for Information and library science, the Pearson correlations between year-1 and year-2 impact levels per journal volume class are quite variable. Apparently is the measurement of citation impact form year-1 to year-2 in this field, as the range of rank correlations is quite wide in the year-1/year-2 block of scores, while this becomes less wide in the year-2/ywear-3 comparison, although the journal volume class of journals with 26–35 publications per year shows a strong decrease first, before the rank correlations start to increase again. In the Journal Subject Category Mathematics (Fig. 2d), the rank correlations between year-1/year-2 fluctuate between 0.69 and 0.92, while the range of rank correlation for the comparison of year-2/year-3 is much more dense, namely ranging from 0.95 to 0.98. The next points of measurement show a trend of increasing correlations from year to year. Finally, in Fig. 2e the Person rank correlations for journal volume classes and their impact in the Journal Subject Category of Pathology are shown. Again, the widest range of correlations is observed for year-1/year-2 comparison, followed by a fast increase of the correlations between the rank positions of the journals in the various journal volume classes.

## Conclusions

This paper presents the results of a study on the development of citation impact over time, and more in particular on the validity of the increasing impact in time, in comparison with short term impact measurement as applied in the impact measurement of the classical Journal Impact Factor. While previously the Journal Impact Factor has been critically analyzed for applying too short citation windows, this paper demonstrates that the conclusion of such invalidity of the length of the citation window was due to a methodological approach, and is not necessarily due to the applied length of citation windows in impact measurement itself.

In our previous studies, we focused on the annual trend of citation impact development, through which we could identify a citation peak. This citation peak was always beyond the citation window applied in the calculation of the classical Journal Impact Factor. This lead to the conclusion that this applied methodology in Journal Impact Factor calculation was wrong. However, if one applies a cumulative method of impact measurement, in which the citation impact of the various years after the year of publication are summed up, we could analyze the validity of the applied citation window from a different perspective.

As we observe citation impact initially to increase, to reach a peak, and then to decrease in volume, this means that the cumulative approach displays a constant increase in citation impact, which reaches a point of saturation at a certain moment. From this, we can analyze the development of citation impact in time based on the rank positions of journals in the various journal volume classes, assuming that we implicitly measure a year to year increase of citation impact. Then, an increase of Pearson correlations from block of years to the next is indicative of the strong resemblance of citation impact development in time.

In this study, we compared the various years of publication with each other. This results in Pearson correlations for every two years of publication, form year-1 to year-7. This study has shown that the Pearson correlations between blocks of publication years are increasing in time, reaching a full 100% in the middle and later years in the analysis. From this observation, of increasing correlation from year to year, from year-2 onwards, we can conclude that citation impact measurement in year-2 is highly predictive of the citation impact reached in later years in the development of citation impact. This leads to the conclusion that Journal Impact Factors are in fact a relatively good predictor of the citation impact of a journal reached in the somewhat longer run.

However, we need to make a few remarks on the results in the study with respect to the conclusion drawn in the previous paragraph. A first remark relates to the comparison of year-1 with year-2 in the citation impact measurement conducted in this study. Obviously, the Pearson correlations observed between year-1 (year of publication) and year-2 are rather weak in some occurrences, and do fluctuate across journal volume classes, while the comparison between journal volume classes in the two publication years 2000 and 2004 is not stable as well. A second remark relates to the journal volume class with the lowest number of publications. Here we observe a slower pace of increasing Pearson correlations from block to block, indicative of more fluctuating citation patterns within that journal volume class, although we finally observe a convergence also in this class towards increasing correlations, thus of a stronger resemblance of the citation development over the years.
